# Stroke Thrombolysis in the Context of a Pituitary Macroadenoma

**DOI:** 10.7759/cureus.55560

**Published:** 2024-03-05

**Authors:** Andrew Evans, Jahanzeb Rehan

**Affiliations:** 1 Stroke Medicine, King's Mill Hospital, Sutton-in-Ashfield, GBR

**Keywords:** ischemic stroke, stroke medicine, stroke treatment, stroke, stroke protocol

## Abstract

The presence of an intracranial tumour is a relative or absolute contraindication to stroke thrombolysis by most guidelines across the world. This is based on the risk of iatrogenic symptomatic intracranial haemorrhage related to the tumour. We present a patient where the decision to proceed with thrombolysis was complicated by an incidental finding of an intracranial tumour. The decision was made to proceed with thrombolysis. The patient had excellent functional recovery in the hours after administration and didn’t suffer any intracranial haemorrhage. The evidence around excluding this patient group from thrombolysis is scant and mostly of low quality. Original randomised controlled trials or stroke thrombolysis excluded this patient group and there have been none since. Published case reports and series are heterogeneous in their conclusions regarding the risk of symptomatic haemorrhage following thrombolysis in patients with intra-axial and extra-axial neoplasms. Further studies may clarify guidelines.

## Introduction

In acute ischaemic stroke, thrombolytic treatment using recombinant tissue plasminogen activator (r-tPA) is now a preferred approach if strict criteria are met, including administration within 4.5 hours of onset of symptoms, and contraindications avoided including exclusion of haemorrhagic stroke and no other recent incidences of ischaemic stroke. Outcomes, defined by recovery and residual disability in the months following an ischaemic stroke, have been shown to be superior in the patient group treated with thrombolysis [1].

Both prior and currently active cancers have been identified as risk factors, increasing the probability of ischaemic stroke [2-4]. A recent review estimates that one in seven to eight patients with ischaemic stroke have a known or hidden cancer [2]. The mechanism of this effect is not fully clear, but it has been found that in 40% of cases, cancer-related coagulopathy may be implicated. Although cancers of the prostate, breast, and colorectum are most commonly found as comorbid in acute ischaemic stroke patients, a primary brain tumour has been identified as carrying one of the highest relative risks of fatal stroke of all types of cancer when compared to the general population [5].

Despite this relatively high prevalence of cancer as a comorbidity in ischaemic stroke, there remains considerable uncertainty as to its implications for the preferred therapeutic strategy. In the specific case of brain cancers, thrombolytic treatment is widely considered to carry a risk of spontaneous cerebral haemorrhage (SCH) if intracranial neoplasms (ICNs) are present, although the evidence for this is not strong [6]. Studies based on limited clinical data have suggested a more serious risk for malignant than benign tumours and that intra-axial tumours present a greater risk than extra-axial [7]. One recent study suggested that only a relatively small additional risk of SCH was associated with most intracranial tumour types, with the possible exception of pituitary tumours, for which - on the evidence of a very small sample - a more serious risk was identified [8]. 

When deciding on whether a patient should receive intravenous thrombolysis, a history of known cancer or an incidentaloma picked up on imaging can therefore present a management dilemma. In practice, most of this patient group who attend our stroke unit do not receive thrombolysis, due to concerns regarding iatrogenic intracerebral haemorrhage (ICH) associated with the tumour, and are managed conservatively with secondary preventative medications and are considered for mechanical thrombectomy should the criteria for this be satisfied.

We present a patient who presented with an acute ischaemic stroke that was managed with intravenous thrombolysis. An incidental finding of a large pituitary macroadenoma complicated the decision to treat with thrombolysis as there is varying opinion as to the degree of contraindication that ICNs present. This patient had an excellent outcome. We also examine the current evidence base for thrombolysis in patients with brain tumours to see the basis of often excluding this patient group from this treatment.

## Case presentation

A man in his mid-60s presented with a witnessed onset of slurred speech, right-sided limb weakness, and right-sided facial droop; he had no prodromal symptoms. He was usually fit and well with no significant past medical history, was not taking any regular medication, and was independent in all activities of daily living. The patient had not lost consciousness at any point before or after the onset of symptoms and had no seizure-typical symptoms such as limb jerking. The man was in the emergency department within two hours of the onset of his symptoms.

Neurological examination revealed moderately reduced power (MRC scale 3/5) in the right arm and leg, right facial droop, dysarthria, and right arm drift. There was no clear sensory or visual field defect. The man’s pupils were equal and reactive. He was oriented to time, place, and person. Cardiorespiratory and abdominal examination was unremarkable. The patient’s observations were all within normal ranges. The National Institutes of Health Stroke Scale (NIHSS) was calculated to be 10 at presentation.

**Figure 1:**
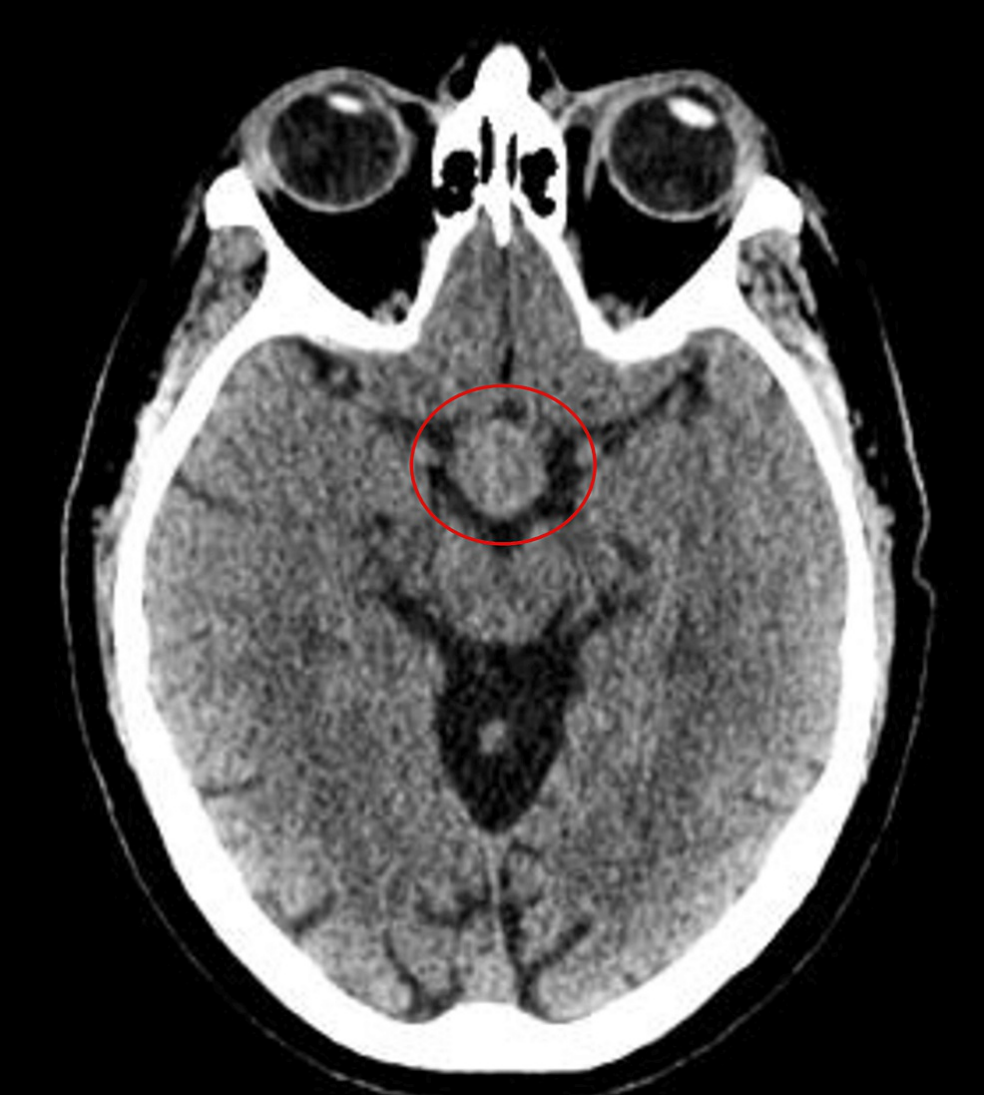
CT brain scan showing no evidence of haemorrhage or ischaemia and a large sellar mass measuring 2.9x2.7x4.1 cm

Given the clinical suspicion of acute stroke, an urgent CT brain was organised to see any evidence of haemorrhage. The scans did not show any evidence of bleeding or any infarcted areas of the brain. However, there was an incidental finding of a large sellar mass. The gentleman had no prior imaging of his brain on record. The mass was reported as most likely being a large pituitary macroadenoma, as seen in Figure 1. Routine blood tests were unremarkable. Capillary blood glucose was 7.0 mmol/L. 

The symptomatology of this patient was in keeping with acute stroke. The CT brain revealed no evidence of haemorrhagic stroke and, as such, our top differential at this point was an acute ischaemic stroke.

It is important to consider stroke mimics in this situation, however, to avoid unnecessary treatments and their associated risk of iatrogenic harm. Metabolic derangements such as hypoglycaemia were ruled out following admission blood test results. The history and examination were not supportive of other common stroke mimics such as migraine or seizures. The moderate NIHSS of 10 was also more in keeping with acute stroke.

A further consideration is the incidental finding of the probable pituitary macroadenoma. Cancers can increase stroke risk through a variety of mechanisms including causing a state of hypercoagulability, tumour embolism, intracranial pressure changes and invasion into or compression of cerebral vasculature [4]. The symptoms in this case, however, were acute onset and damage to the area of the brain where the lesion was identified on CT imaging was felt to be unlikely to account for this man’s symptoms. Interestingly, there was no clear visual field defect as one might expect with a large sellar lesion.

In the absence of clear guidelines and evidence in these circumstances, with clear acute stroke symptoms, the decision was made, with the patient’s agreement, to proceed with thrombolysis. The patient received a bolus injection of alteplase (r-tPA) followed by an infusion as per local guidelines. The treatment was started within four hours of the onset of symptoms. The patient was moved to the stroke unit for further assessment. This patient presented on a weekend when there was no provision for mechanical thrombectomy, a service offered by a nearby tertiary centre hospital which at the time of writing does not offer 24-hour weekend service to our district general hospital. As such, a CT angiogram and CT perfusion scans were not performed as they would not have changed the patient's management. 

**Figure 2:**
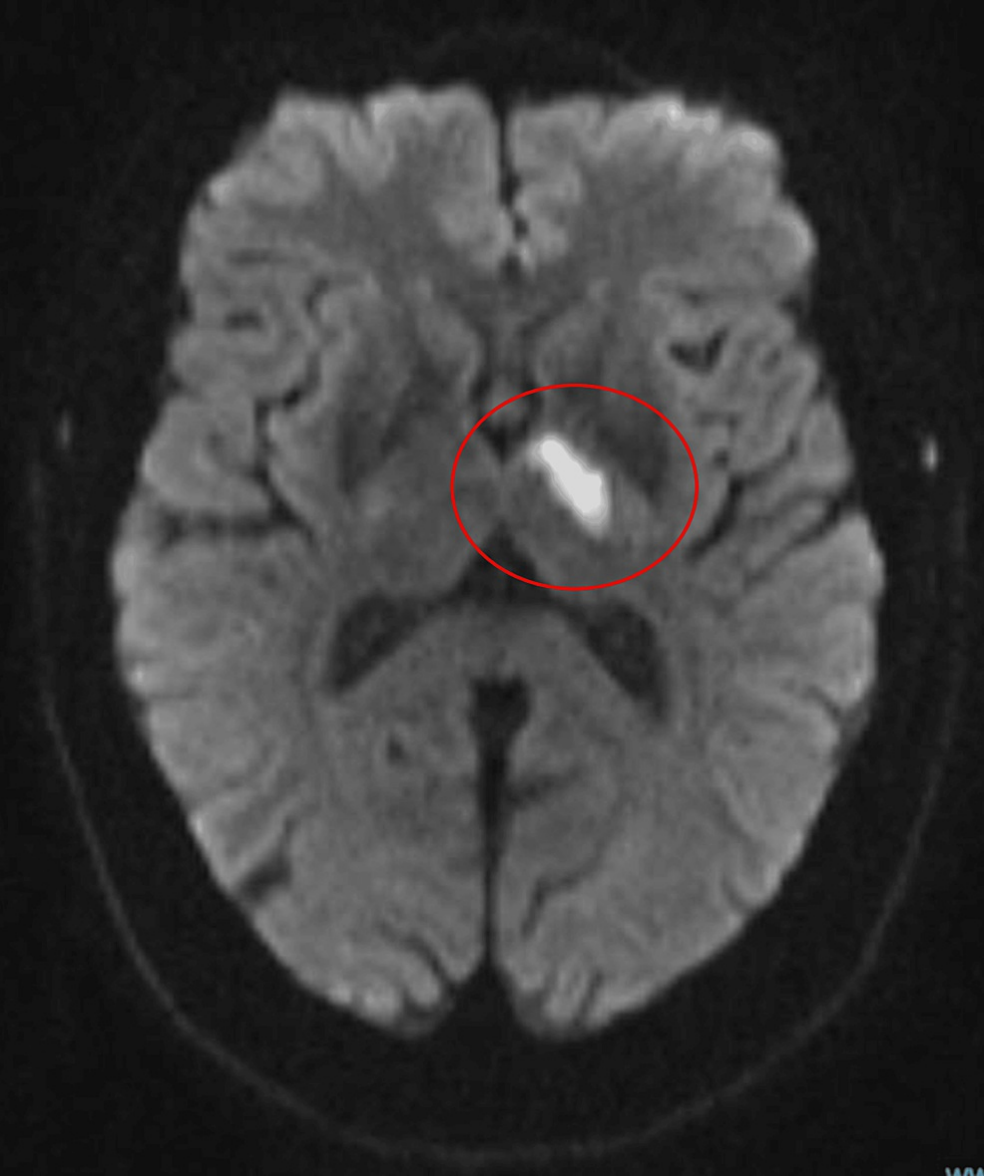
MRI brain confirming acute left basal ganglia ischaemic stroke

**Figure 3:**
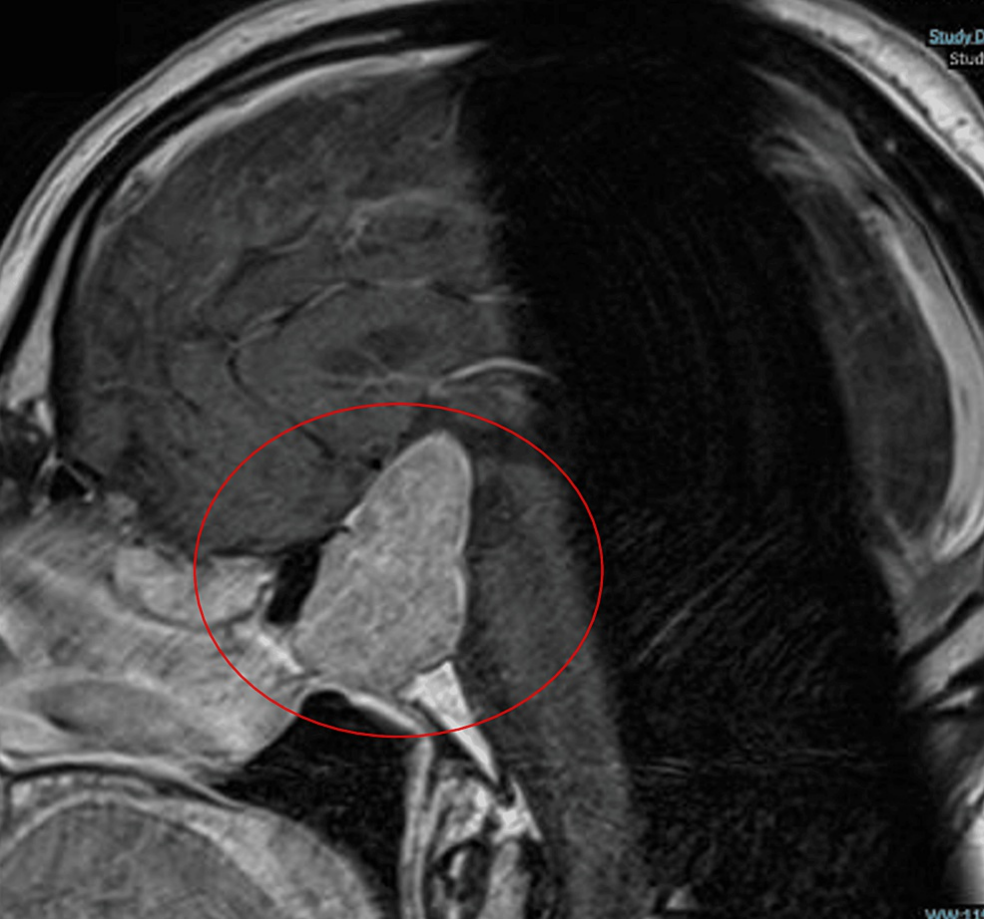
MRI brain showing a large pituitary macroadenoma

The patient’s post-thrombolysis NIHSS decreased to 4 a few hours later. No 24-hour post-thrombolysis CT brain scan was performed but a subsequent MRI brain revealed an acute left basal ganglia infarct, as seen in Figure 2. The MRI scan also confirmed the pituitary macroadenoma, as seen in Figure 3. 

The patient was referred to local Endocrine and Neurosurgical services and listed with the pituitary multi-disciplinary team (MDT). He was placed on mineralocorticoid therapy after being found to be in a state of hypoadrenalism. At the time of writing, the patient is awaiting the MDT outcome for management options of the pituitary lesion. Interestingly, a formal ophthalmology review found no visual field defect or visual impairment.

The patient was discharged by our therapy teams as his mobility had improved greatly from admission. A review by our speech and language therapy team found mild dysarthria but no evidence of dysphagia or dysphasia. The patient remained on the stroke unit for three days before being discharged, having been established on secondary preventative medications. The patient walked from the ward and their speech had greatly improved. 

The patient returned to the stroke clinic approximately three months later. He had no significant residual symptoms, his weakness had almost completely resolved, and he felt that his speech was almost completely back to normal. We felt that this represented an excellent outcome for this patient. The aetiology of the stroke remains unclear, and outpatient investigations including cardiac arrhythmia monitoring have been unremarkable.

## Discussion

ICNs being listed as a contraindication in guidelines appear to be based on rather little evidence but rather to be a “sensible precaution”, given the well-recognised increased risk of SCH associated with thrombolysis. In reality, the magnitude of the risk of symptomatic ICH in patients with an ICN who receive thrombolysis is unclear. Because of a conventional reluctance to use thrombolysis in these patients, data for the incidence and relative risk of symptomatic and/or fatal ICH are scarce in the literature. The National Institute of Neurological Disorders and Stroke rt-PA Stroke Study Group trial in 1996 was the basis for the adoption of r-tPA into preferred practice [9]. Patients with brain tumours were not included explicitly in this trial, and there have been no further randomised-control trials since that we are aware of. Data on the risk of symptomatic and fatal ICH following thrombolysis for this patient group are limited to case reports and case series.

A systematic review of case reports of patients with intracranial tumours who received stroke thrombolysis from 2014 found that patients with benign brain tumours had a risk of ICH that was no higher than acute ischaemic stroke patients who did not have a brain tumour but that patients with malignant brain tumours tended to have poorer outcomes generally and a higher relative risk of ICH following thrombolysis [10]. The authors concluded that thrombolysis might be considered in extra-axial benign-appearing neoplasms (e.g., meningioma) but is not advisable in intra-axial primary or metastatic neoplasm. A 2015 analysis of in-hospital outcomes reached a similar conclusion, reporting that neoplasms were associated with a higher mortality risk if they were intra-axial rather than extra-axial [7]. A 2021 critical appraisal of the evidence noted that the presence of intra-axial intracranial tumours represents an absolute contraindication to thrombolysis while acknowledging that this conclusion is based only on limited case series or case reports [11]. They further stated that extra-axial brain tumours should not preclude patients with symptoms of acute cerebral ischaemia from receiving thrombolysis.

A series of 104 cases reported in 2023 found somewhat different results [8]. The frequency of intracranial haemorrhage for patients who received thrombolysis and who had identified intracranial tumours was found to be within the range measured for patients without a brain tumour but toward the top end of it, suggesting that there is a small but not negligible additional risk associated with cancer. In this case, though, they did not find a greater risk associated with intra-axial tumours. Notably, however, they identified pituitary tumours (an extra-axial tumour) as potentially being associated with a significantly greater risk, although this was based on a sample of only four patients, two of whom suffered symptomatic haemorrhage following thrombolysis. The paper also searched for correlations with various parameters, including tumour size and ICH risk, and found no significant relationship within their small data set.

Despite these various investigations of the issue, no consensus has emerged as to whether and in what circumstances thrombolysis should be used in ischaemic stroke cases when ICNs are present. The case series and reports are clearly at risk of being influenced by selection bias, the sample sizes tend to be small, and the results are not entirely consistent. It would, therefore, be inappropriate to draw firm conclusions from these studies and this has undoubtedly contributed to the inconsistency and vagueness often found in guidelines for the use of thrombolysis in treating ischaemic stroke when a brain tumour is present.

The guidelines published by the American Heart Association /American Stroke Association now recommend that thrombolysis should be used to treat acute ischaemic stroke in the presence of extra-axial ICNs but is contraindicated when there are intra-axial ICNs [12]. The decision to proceed with thrombolysis in the case we have described would be supported by these guidelines. However, this is not always reflected in guidelines published elsewhere. The European Stroke Association guidelines state simply that cancer should not be an absolute contraindication against thrombolytic treatment, although caution seems appropriate [13]. In the UK, individual trust guidelines do not always make clear recommendations and sometimes state that all intracranial neoplasms are an absolute contraindication for thrombolysis. This is often also the “safety first” view taken by practising clinicians in the absence of clear guidelines. This heterogeneity reflects the current reality that there are still too few clear and high-quality data to determine unequivocally the relative risk of ICH in the presence of intracranial tumours based on type, size, or location. It remains imperative to continue to have frank discussions of the risks and benefits of this treatment with the patient, however, in the absence of clear data, it is not possible for a patient to make a fully informed decision. Further high-quality investigations of the outcomes for this patient group following stroke thrombolysis are needed to allow a clear, unambiguous consensus policy to be formulated and validated.

## Conclusions

There is heterogeneity in the literature regarding intravenous r-tPA being contraindicated for patients with intracranial tumours; there are differing conclusions from the existing small studies regarding the risk of haemorrhage following stroke thrombolysis for this patient group. High-quality prospective studies are needed.

Extra-axial and benign brain tumours may be associated with a similar risk of symptomatic or fatal ICH following thrombolysis as acute ischaemic stroke patients without ICNs, but the haemorrhagic risk may be higher with intra-axial or malignant tumours. However, existing data are insufficient to establish these distinctions clearly. 
